# Imaging Characteristics of Colloid Cysts: A Case Series Correlating Cyst Size and Location With Associated Acute Hydrocephalus

**DOI:** 10.7759/cureus.98691

**Published:** 2025-12-08

**Authors:** Ravichandra Varma S, Rahul Suresh, Jeevika Ujjappa

**Affiliations:** 1 Radiodiagnosis, Jagadguru Jayadeva Murugarajendra Medical College, Davangere, IND

**Keywords:** colloid cyst, high-risk zones, hydrocephalus, mri, third ventricle

## Abstract

This retrospective study evaluates the diagnostic role of CT and MRI in identifying colloid cysts and assessing their association with hydrocephalus. Data from 30 patients diagnosed with third ventricular colloid cysts at Jagadguru Jayadeva Murugarajendra Medical College, Davangere, between 2017 and 2024 were reviewed. Imaging characteristics, including cyst size, location, and potential complications such as hydrocephalus, were analyzed. Colloid cysts larger than 10 mm, located in high-risk regions, and observed in younger patients were more likely to develop complications, including acute hydrocephalus. Asymptomatic cysts exceeding 10 mm in high-risk zones warrant regular imaging follow-up and timely surgical intervention, if necessary, to prevent sudden clinical deterioration. The findings highlight MRI’s superiority in demonstrating signal characteristics, cyst contents, and subtle variations, making it valuable for planning management strategies. Overall, larger cyst size and high-risk anatomical positioning strongly correlate with symptomatic presentation and complications, emphasizing the importance of early identification, imaging-based risk stratification, and vigilant monitoring to prevent catastrophic outcomes.

## Introduction

Colloid cysts are benign, slow-growing lesions located in the anterior third ventricle, most commonly near the foramen of Monro, an anatomically critical region for CSF circulation [1,2]. Although histologically nonneoplastic and frequently asymptomatic, their strategic location can lead to intermittent or persistent obstruction of CSF flow, resulting in ventriculomegaly, elevated intracranial pressure (ICP), or acute obstructive hydrocephalus [3,4]. In rare cases, rapid neurological deterioration and sudden death have been reported [5]. Clinical presentation ranges from incidental radiologic findings to acute neurological emergencies, underscoring the importance of early identification to prevent irreversible outcomes.

Colloid cysts account for approximately 0.5-2% of intracranial tumors and are most commonly identified in young to middle-aged adults, although they may occur at any age [1-3]. Histologically, they consist of epithelial-lined cystic structures containing viscous gelatinous material composed of cholesterol crystals, mucopolysaccharides, and proteinaceous debris [6]. Despite their benign nature, their proximity to the foramen of Monro can result in clinically significant CSF obstruction and potentially catastrophic consequences [3,5].

The pattern of obstruction may vary. Some cysts exhibit a “ball-valve” mechanism, producing episodic or positional symptoms [4,5]. Symptomatic patients may present with persistent headache, nausea, vomiting, gait imbalance, visual disturbances, memory deficits, or altered sensorium. Acute obstruction of CSF pathways may result in severe hydrocephalus, coma, herniation, or death if intervention is delayed [2,4]. Even when detected incidentally, lesions displaying high-risk imaging features require close clinical and radiologic surveillance.

Neuroimaging is central to evaluation and risk stratification. CT typically demonstrates hyperdense or isodense intraventricular nodules, while MRI provides superior soft-tissue contrast, allowing detailed characterization of cyst contents, ventricular enlargement, and secondary signs of CSF flow disruption, such as periventricular edema or transependymal migration [6-8]. Imaging findings also permit classification of cysts into anatomical risk zones around the foramen of Monro, which are clinically relevant to the likelihood of obstructive hydrocephalus. Because acute clinical deterioration is often predicted by imaging parameters rather than cyst size alone, establishing objective radiologic predictors is essential.

To address this clinical need, the present study evaluates the association between two measurable neuroimaging parameters, cyst size and anatomical location within ventricular risk zones, and the presence of acute hydrocephalus at presentation. Acute hydrocephalus was defined as an Evans Index ≥0.30 and/or radiologic evidence of transependymal CSF flow. The unit of analysis was individual patients, and a prespecified cyst size threshold of >10 mm was applied based on literature and clinical practice relevance. This retrospective case series summarizes seven years of institutional experience from a tertiary care center and highlights imaging-based predictors of clinically significant CSF obstruction.

## Materials and methods

This retrospective case series was conducted at Jagadguru Jayadeva Murugarajendra Medical College, Davanagere, to evaluate the imaging characteristics of colloid cysts. Medical records from January 2017 to December 2024 were systematically reviewed to identify patients with radiologically confirmed colloid cysts who underwent neuroimaging. A total of 30 eligible cases were included. CT and MRI studies were analyzed to assess cyst size, anatomical location, impact on CSF flow, and associated ventricular changes. The objective was to correlate imaging parameters with clinically significant obstruction and provide insights into diagnostic interpretation, disease progression, and implications for treatment planning.

Inclusion and exclusion criteria for patient selection

Patients were included if they were diagnosed with a third ventricular colloid cyst based on MRI and/or CT imaging during the study period and had complete clinical and imaging data available for review and analysis. Patients were excluded if their imaging records were incomplete or if the cystic lesions were not consistent with a colloid cyst (e.g., lesions of noncolloid origin).

Data collection and imaging assessment

Demographic, clinical, and neuroimaging data were retrospectively extracted from hospital records. Imaging parameters were systematically assessed for each patient:

Cyst Size

Maximum cyst diameter was measured in millimeters on CT or MRI as the largest linear dimension in either the axial or sagittal plane, using digital calipers on the PACS workstation.

Cyst Location

Anatomical position within the third ventricle was categorized according to radiologically defined ventricular zones relative to the foramen of Monro: Zone I (pre-foraminal/superior), situated anterior or superior to the foramen of Monro, typically abutting the roof of the third ventricle; Zone II (foraminal/central), occupying the foramen of Monro region, bordered anteriorly by the anterior commissure and posteriorly by the column of the fornix; and Zone III (post-foraminal/inferior), extending inferiorly into the third ventricle toward the hypothalamic or infundibular recess. This classification was applied uniformly and recorded for each patient.

Complications/Ventricular Effects

Hydrocephalus was defined a priori as an Evans Index ≥0.30 and/or the presence of transependymal CSF flow on MRI or periventricular lucency on CT. Ventriculomegaly that did not meet hydrocephalus criteria was recorded separately. Additional imaging signs of CSF flow obstruction, such as ballooning of the foramen of Monro or disproportionate enlargement of the lateral ventricles relative to the third ventricle, were also noted.

Statistical analysis

This retrospective case series included 30 patients with radiologically confirmed third ventricular colloid cysts. Clinical and imaging parameters were systematically analyzed to assess associations between cyst characteristics, including maximal cyst diameter and anatomical location, and the presence of acute hydrocephalus. Patients were categorized into two groups: symptomatic (with hydrocephalus) and asymptomatic (without hydrocephalus), and key demographic and imaging variables were compared to identify potential predictors of symptomatic presentation.

All data were tabulated in Microsoft Excel and analyzed using IBM SPSS Statistics for Windows, Version 25.0 (Released 2017; IBM Corp., Armonk, NY, USA). Categorical variables were summarized as frequencies and percentages. Associations between categorical variables were tested using the chi-square test, with Fisher’s exact test applied when expected cell counts were <5. A p-value <0.05 was considered statistically significant, and a p-value <0.01 was considered highly statistically significant.

## Results

Demographic analysis of symptomatic vs. asymptomatic patients

**Figure 1:**
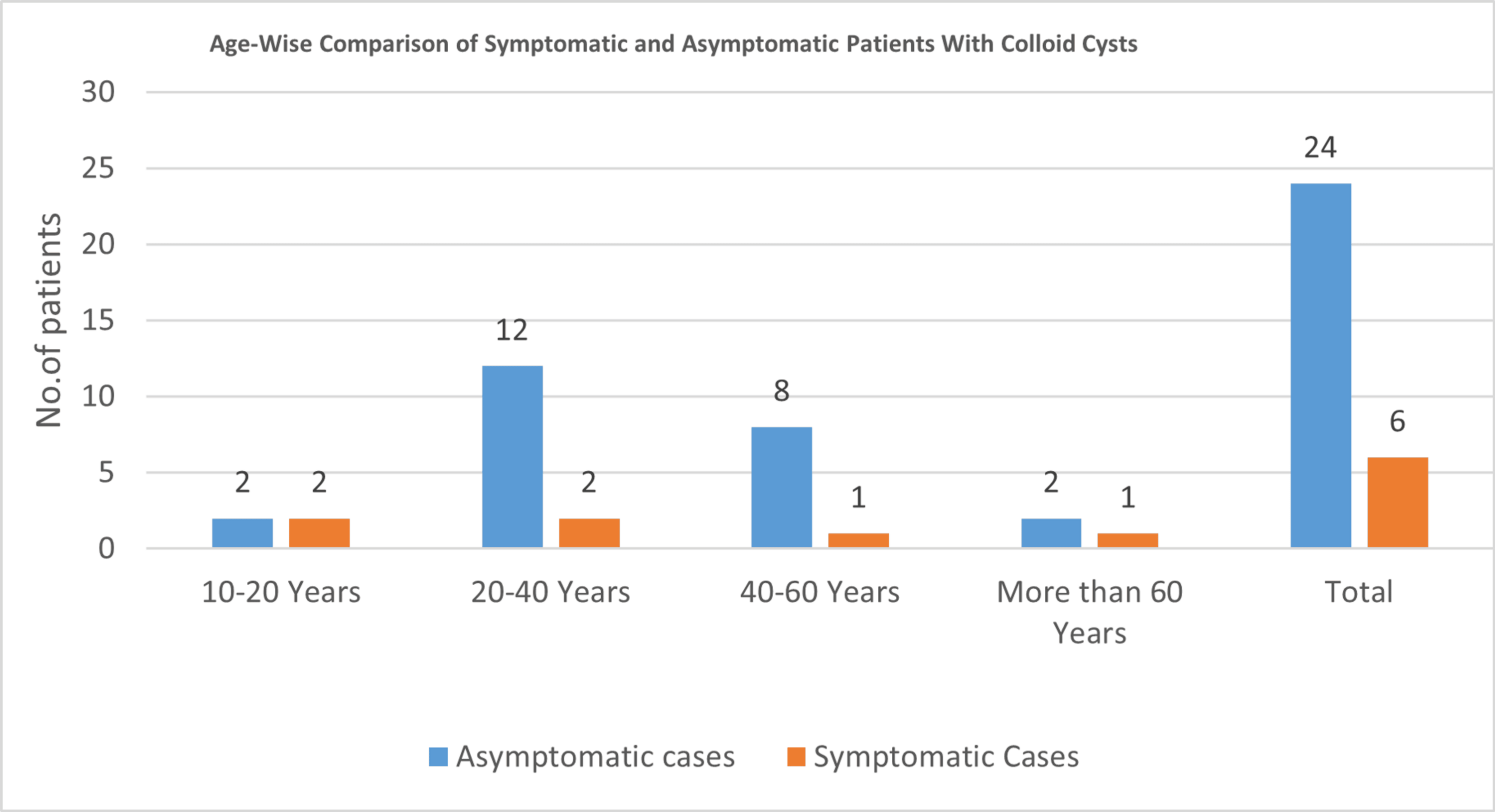
Age distribution of symptomatic and asymptomatic patients with colloid cysts

Patient demographics (Table 1) were analyzed to assess the distribution of symptomatic (with hydrocephalus) and asymptomatic (without hydrocephalus) colloid cyst cases across different age groups and genders. No statistically significant gender-based difference was observed (χ² = 0.27, p = 0.60). A higher proportion of symptomatic cases was noted in the 20- to 40-year age group, although this association did not reach statistical significance (χ² = 2.41, p = 0.30). As illustrated in Figure 1, symptomatic cases were more concentrated within this age range, whereas asymptomatic cases showed a broader distribution, with peaks in both the 20-40 and 40- to 60-year age groups. Although not conclusive, this trend suggests a possible age-related predisposition that warrants further evaluation in larger cohorts.

**Table 1:** Age and gender distribution of symptomatic and asymptomatic patients with colloid cysts (n = 30)

Age group	Total patients	F	M	Symptomatic (hydrocephalus)		Asymptomatic (no hydrocephalus)	
				F	M	F	M
10-20 years	4	2	2	1	1	1	1
20-40 years	14	6	8	1	1	5	7
40-60 years	9	5	4	1	0	4	4
>60 years	3	1	2	0	1	1	1
Total	30	14	16	3	3	11	13

Analysis of cyst size and symptom development

**Figure 2:**
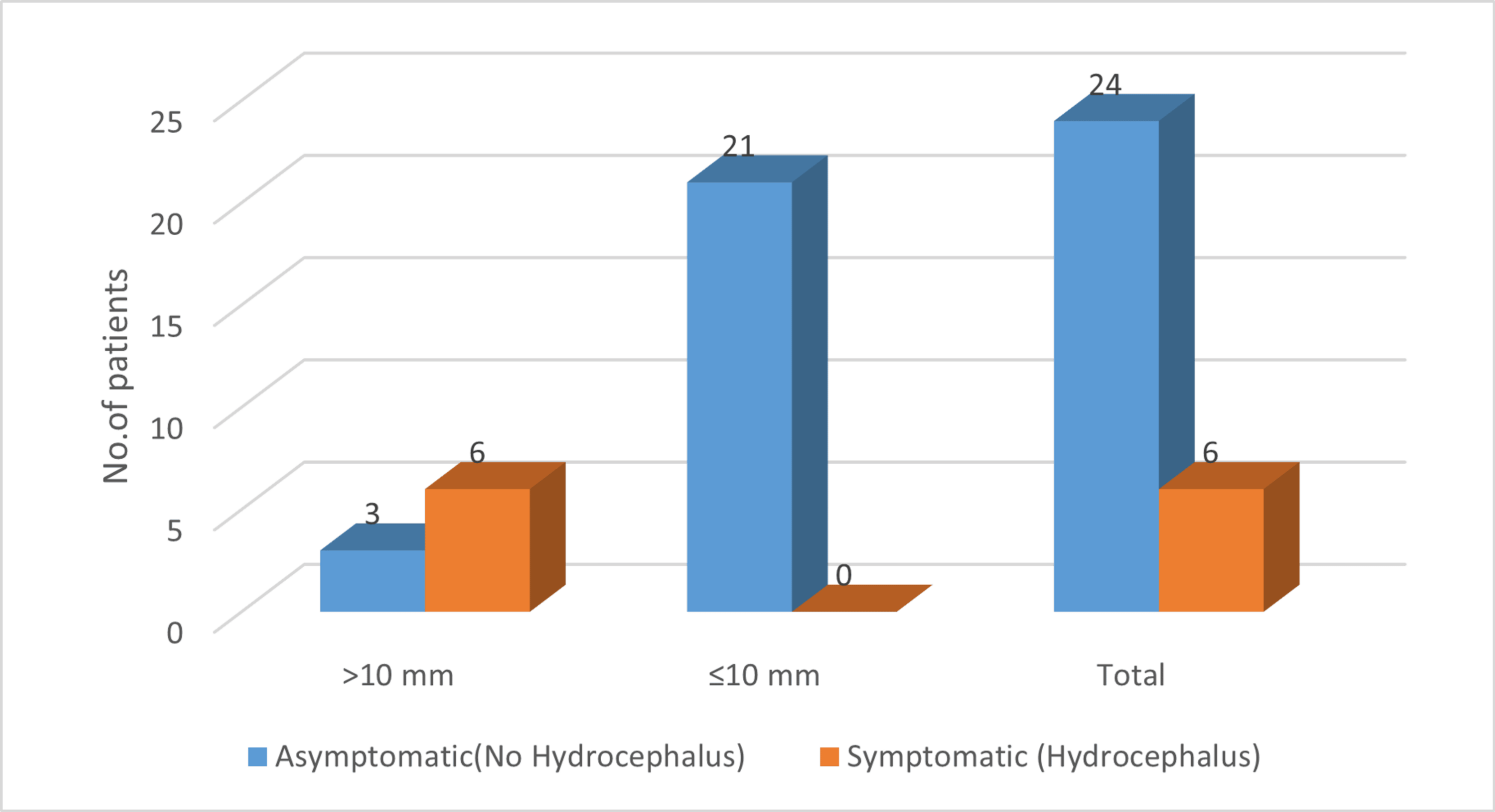
Cyst size distribution and its association with symptom development

The relationship between cyst size and symptom development was analyzed (Table 2). A chi-square test demonstrated a statistically significant association between cysts larger than 10 mm and the presence of hydrocephalus. Among patients with cysts >10 mm (n = 9), six (66.7%) were symptomatic, whereas none of the patients with cysts ≤10 mm (n = 21) developed hydrocephalus. This association was statistically significant (χ² = 14.29, p < 0.001), indicating that larger cysts were more likely to be symptomatic and associated with obstructive hydrocephalus. Figure 2 illustrates this distribution, showing that all symptomatic cases involved cysts >10 mm, while all patients with cysts ≤10 mm remained asymptomatic.

**Table 2:** Distribution of cyst size in relation to symptom development

Cyst size	No. of patients (N)	Symptomatic (hydrocephalus)	Asymptomatic (no hydrocephalus)
>10 mm	9	6	3
≤10 mm	21	0	21
Total	30	6	24

Analysis of cyst location and symptom development

**Figure 3:**
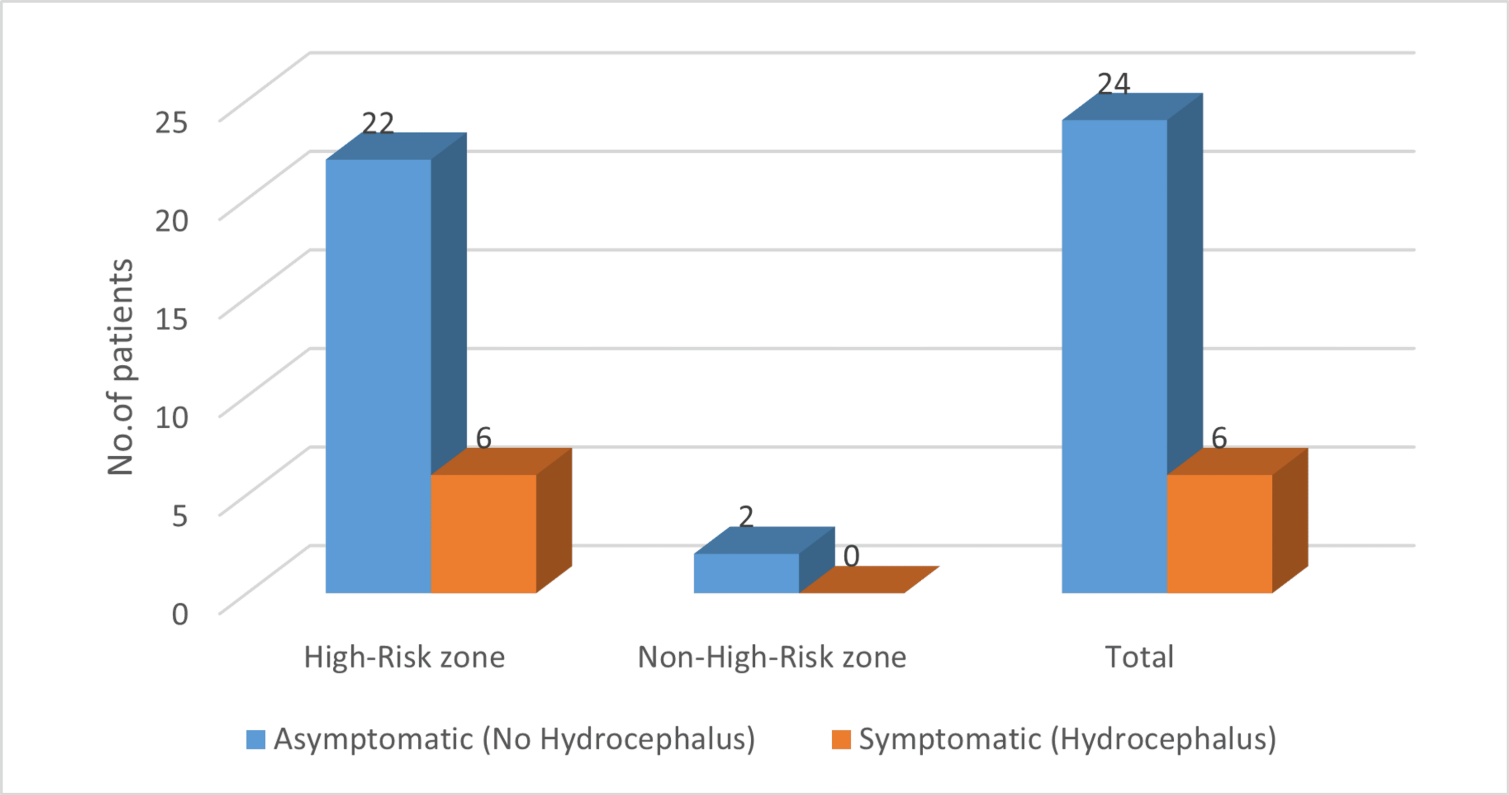
Cyst location by risk zone and its association with symptom development

Cyst location was classified into high-risk and non-high-risk zones based on anatomical criteria (Table 3). Due to the small sample size in the non-high-risk group (n = 2), Fisher’s exact test was applied. Among the 28 patients with cysts in high-risk zones, six (21.4%) were symptomatic, whereas both patients with cysts in non-high-risk zones were asymptomatic. The association between cyst location and symptom development did not reach statistical significance (Fisher’s exact test = 0.82; p = 0.24). However, the observation that all symptomatic patients had cysts located in high-risk anatomical zones suggests a potential influence of cyst location on clinical presentation. Although not statistically conclusive, this trend may warrant further investigation in larger cohorts. Figure 3 illustrates this distribution.

**Table 3:** Distribution of cyst location by risk zone and its association with symptom development

Location	No. of patients (N)	Symptomatic (hydrocephalus)	Asymptomatic (no hydrocephalus)
High-risk zone	28	6	22
Non-high-risk zone	2	0	2
Total	30	6	24

Representative cases

Case 1

A 34-year-old female presented to the emergency department with a sudden-onset severe headache, multiple episodes of vomiting, and transient loss of consciousness. There was no prior history of seizures, trauma, or neurological illness.

**Figure 4:**
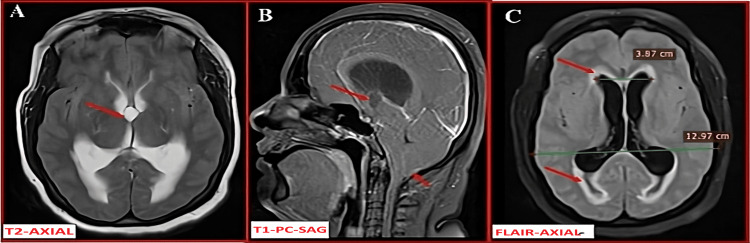
MRI brain images: (A) axial T2-weighted, (B) sagittal post-contrast T1-weighted, and (C) axial FLAIR images (A) Axial T2-weighted image shows a well-defined hyperintense cystic lesion at the roof of the third ventricle (red arrow), obstructing the foramen of Monro. (B) Sagittal post-contrast T1-weighted image demonstrates the non-enhancing nature of the lesion (red arrow) and inferior descent of the cerebellar tonsils below the foramen magnum, consistent with tonsillar herniation. (C) Axial FLAIR image reveals periventricular CSF ooze and dilated bilateral lateral ventricles (red arrows), suggestive of acute hydrocephalus (Evans index: 0.3). Note: All images are original and obtained from the institutional study dataset. FLAIR, fluid-attenuated inversion recovery

MRI revealed a third ventricular colloid cyst with acute obstructive hydrocephalus (Figure 4).

Case 2

A 50-year-old male presented with a sudden-onset severe headache and repeated vomiting. Neurological deterioration was noted on admission, with reduced responsiveness and signs of increased ICP. There was no prior history of neurological illness or trauma.

**Figure 5:**
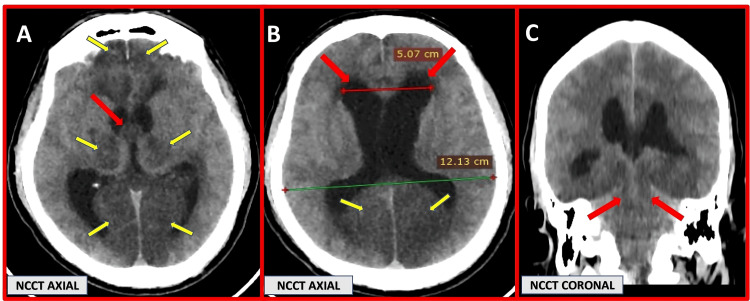
NCCT brain images: (A and B) axial sections and (C) coronal reformatted image (A) Axial NCCT shows a hypodense cystic lesion at the roof of the third ventricle (red arrow), measuring approximately 13 × 11 mm (HU: 12), obstructing the foramen of Monro. Acute infarcts are noted in the bilateral thalami and frontoparietal lobes (yellow arrows). (B) Axial CT image demonstrates marked dilatation of the lateral ventricles (Evans index: 0.4) with additional acute infarcts in the bilateral parietal lobes (yellow arrow). (C) Coronal CT section reveals central transtentorial herniation (red arrow) and diffuse cerebral edema. Note: All images are original and obtained from the institutional study dataset. NCCT, non-contrast CT

CT imaging revealed a third ventricular colloid cyst with acute obstructive hydrocephalus and associated acute complications (Figure 5).

Case 3

A 16-year-old boy presented with a sudden loss of consciousness. On arrival, he was unresponsive and exhibited signs of increased ICP. There was no history of preceding trauma, seizures, or known neurological conditions.

**Figure 6:**
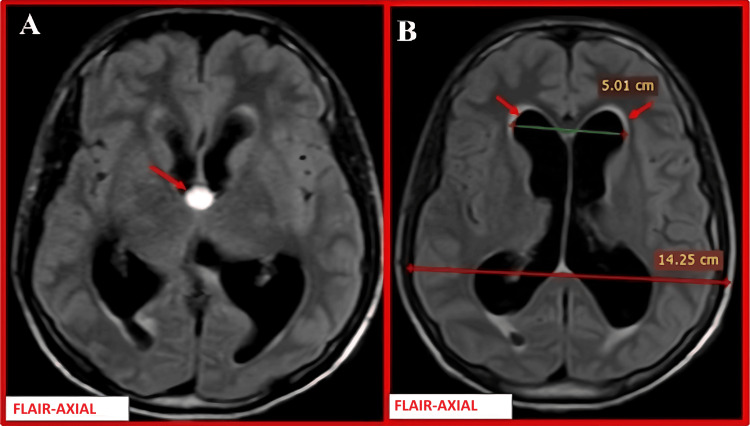
MRI brain images in (A and B) axial FLAIR sections (A) Axial FLAIR image shows a hyperintense cystic lesion at the roof of the third ventricle (red arrow), measuring approximately 12 × 11 × 14 mm, obstructing the foramen of Monro. (B) Axial T2-weighted image demonstrates dilated bilateral lateral ventricles (red arrows) with an Evans index of 0.35, consistent with acute obstructive hydrocephalus. Note: All images are original and obtained from the institutional study dataset. FLAIR, fluid-attenuated inversion recovery

MRI revealed a third ventricular colloid cyst with acute obstructive hydrocephalus (Figure 6).

Case 4

A 22-year-old female underwent non-contrast CT (NCCT) brain imaging for evaluation of intermittent giddiness. Neurological examination was unremarkable, with no signs of increased ICP or focal deficits.

**Figure 7:**
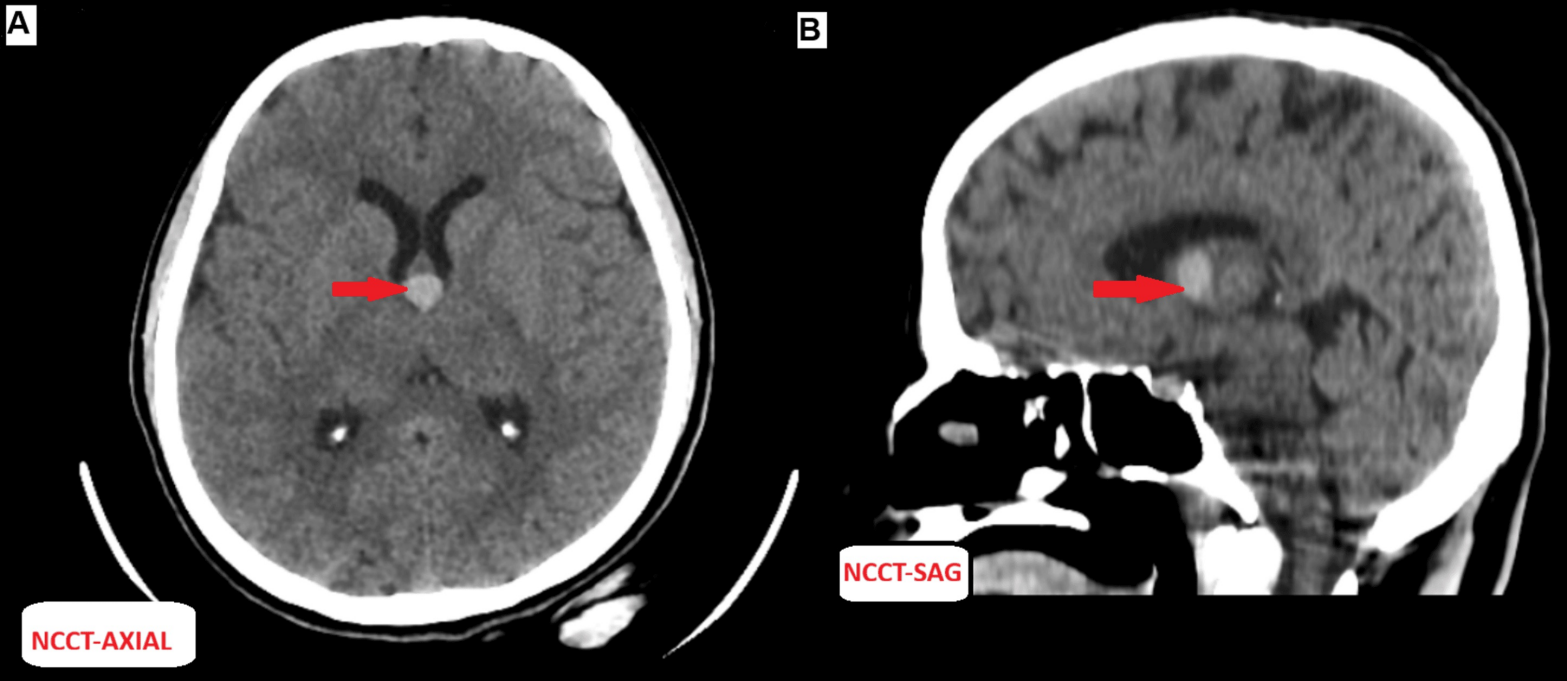
NCCT brain images: (A) axial section and (B) sagittal reformatted image (A) Axial NCCT shows a well-defined hyperdense cystic lesion near the foramen of Monro (red arrow), measuring approximately 9.4 × 9.0 mm (HU: 47). (B) Sagittal section confirms the lesion’s location, with no associated hydrocephalus or CSF flow obstruction, consistent with an incidental finding. Note: All images are original and obtained from the institutional study dataset. NCCT, non-contrast CT

CT findings revealed an incidentally detected third ventricular colloid cyst (Figure 7).

These cases illustrate the varied clinical presentations of third ventricular colloid cysts, ranging from incidental findings to life-threatening hydrocephalus. Larger cysts (>10 mm) and those located near the foramen of Monro were consistently associated with symptomatic obstruction. This underscores the importance of detailed imaging evaluation to identify high-risk cysts, which may benefit from closer surveillance or early surgical intervention, even in asymptomatic individuals.

Summary

Colloid cysts larger than 10 mm in diameter, particularly those located in high-risk zones such as the anterior third ventricle near the foramen of Monro, are more likely to cause complications, including obstructive hydrocephalus, especially in younger patients. Even when asymptomatic, cysts exceeding 10 mm in these high-risk areas warrant consideration for early surgical intervention to prevent potentially life-threatening outcomes. Conversely, asymptomatic colloid cysts smaller than 10 mm but located in critical regions should be closely monitored with periodic imaging, as they still carry a risk of sudden obstruction and clinical deterioration.

## Discussion

Colloid cysts of the third ventricle are rare, benign, epithelium-lined lesions most commonly located at the anterior roof of the third ventricle, typically near the foramen of Monro [1]. Although histologically nonaggressive, their strategic location poses a significant clinical risk, as even small cysts can obstruct CSF flow and lead to acute obstructive hydrocephalus [2]. Such obstruction may result in elevated ICP, altered sensorium, rapid neurological deterioration, and, in some cases, sudden death [3]. Several studies have documented life-threatening presentations, including abrupt loss of consciousness and uncal herniation, highlighting the potential lethality of untreated or unrecognized colloid cysts [4].

The risk of acute decompensation depends on both cyst size and anatomical location. In our study, cysts larger than 10 mm were significantly associated with hydrocephalus. This finding aligns with prior reports indicating that cysts >10 mm are more likely to impede CSF flow at the foramen of Monro, resulting in ventricular dilatation and increased ICP [5,6]. However, cyst size alone may not fully predict symptom development. Small colloid cysts (<10 mm) have occasionally caused acute symptoms, likely due to positional dynamics or sudden blockage of the CSF pathway [7]. Conversely, large colloid cysts have been reported as incidental findings in asymptomatic individuals, underscoring the variability in clinical behavior [8,9].

The anatomical position of colloid cysts within the third ventricle plays a crucial role in determining clinical risk. Lesions located in Zone I (adjacent to the foramen of Monro) and Zone III (posterior third ventricle) are more likely to obstruct CSF flow, whereas those in Zone II (central region) are less frequently symptomatic [5,8]. Although our study did not demonstrate a statistically significant association between cyst location and symptoms, all symptomatic cases involved cysts situated in high-risk zones (Table 4), a finding consistent with previous reports [9,10].

**Table 4:** Anatomical zones of colloid cysts and associated risk of hydrocephalus Table recreated based on data from Algin et al. and Beaumont et al. [6,9].

Zone	Anatomical location	Hydrocephalus risk	Clinical significance
Zone I	Anterior to the mammillary bodies and massa intermedia	High	Obstructs the foramen of Monro → CSF accumulation and hydrocephalus
Zone II	Between Zones I and III	Low	Less likely to obstruct CSF flow; often asymptomatic
Zone III	Extending to the cerebral aqueduct of Sylvius	High	May obstruct the aqueduct → CSF accumulation and hydrocephalus

Neuroimaging is essential for diagnosis, risk assessment, and surgical planning. On NCCT, colloid cysts typically appear hyperdense or isodense relative to the surrounding brain parenchyma, depending on internal contents such as cholesterol, mucin, protein, or blood products [6,10]. On MRI, these lesions frequently appear hyperintense on T1-weighted sequences, reflecting high protein or lipid content. T2-weighted and fluid-attenuated inversion recovery (FLAIR) signals are variable, ranging from hypointense to hyperintense, depending on viscosity and internal chemical composition [11,12]. Occasionally, colloid cysts may demonstrate atypical radiologic features, including peripheral enhancement, internal hemorrhage, or mixed signal characteristics, which can mimic neoplasms or other cystic masses [13,14]. The imaging features are summarized in Table 5.

**Table 5:** Imaging characteristics of colloid cysts on CT and MRI Table recreated based on data from Algin et al. [6].

Modality	Parameter	Findings
Neuroimaging	Typical location	Anterior third ventricle, at or near the foramen of Monro
Neuroimaging	Morphology	Well-circumscribed, unilocular lesion
CT	Attenuation	Commonly hyperdense; lesions may also appear isodense or hypodense in less frequent cases
	Calcification	Rare; may be present as punctate or peripheral foci
	Contrast enhancement	Typically minimal or absent; occasional thin peripheral rim enhancement reported
MRI	T1-weighted signal intensity	Variable: approximately 50% are hyperintense; others demonstrate iso- or hypointensity depending on cyst content (e.g., protein and cholesterol)
	T2-weighted signal intensity	Heterogeneous: often hypointense due to viscous, proteinaceous fluid. Some lesions may exhibit a central low signal with peripheral hyperintensity, or appear homogeneously hyperintense
	FLAIR imaging	Signal may be suppressed or resemble CSF, potentially obscuring the lesion
	Post-contrast enhancement	Generally absent or minimal; if present, typically appears as a thin rim or enhancement of adjacent vasculature

Histopathologically, colloid cysts are lined by cuboidal or columnar epithelium and filled with gelatinous material composed of mucopolysaccharides, cholesterol crystals, and hemosiderin [14]. Immunohistochemical and ultrastructural studies suggest an endodermal origin, although their embryologic derivation remains debated [15]. This diversity in epithelial lining and cyst contents contributes to the broad spectrum of imaging appearances [14,15].

The natural history of colloid cysts is variable. While some remain indolent for years, others progress rapidly. Asymptomatic cysts are increasingly detected incidentally with the widespread use of MRI. Longitudinal studies indicate that many such lesions remain stable or grow very slowly over time [8,9]. Nevertheless, reports of sudden deterioration, even in previously asymptomatic patients, support early surgical intervention in selected cases [3,4,16].

Management decisions must balance surgical risk against the potential for acute neurological deterioration. Emergent intervention is warranted in patients with symptomatic hydrocephalus or rapid clinical decline, often necessitating CSF diversion procedures such as external ventricular drainage or endoscopic third ventriculostomy [4,17]. Definitive management options include microsurgical resection via transcallosal or transcortical approaches and endoscopic aspiration or excision [10,17,18]. Endoscopic surgery is increasingly favored due to its minimally invasive nature and shorter recovery time, although complete excision may be challenging in some cases. Microsurgical resection allows for more complete removal but carries risks such as forniceal injury and memory impairment [18].

An important yet less discussed aspect of colloid cysts is their potential familial occurrence. Reports of familial colloid cysts suggest a genetic component, although no causative gene has been definitively identified [11-13]. A positive family history should prompt screening of first-degree relatives, particularly if symptoms suggest raised ICP or subtle memory changes. Several familial cases in the literature underscore the relevance of genetic counseling and early imaging in such contexts [12].

Our study highlights the importance of detailed imaging analysis, including both cyst size and location, in predicting symptomatic presentation. While a statistically significant association between location and hydrocephalus was not observed, anatomical position remains clinically relevant. The variability in clinical progression reinforces the need for individualized decision-making based on risk stratification.

Future multicenter prospective studies are needed to better define predictors of rapid clinical decline, optimize the timing of surgical intervention, and explore the role of molecular genetics in familial cases. Incorporating advanced MRI techniques, such as diffusion-weighted imaging and spectroscopy, may further improve diagnostic confidence and risk prediction.

Limitations

This study has several limitations. As a retrospective analysis, it is subject to inherent selection and documentation biases. The relatively small sample size (n = 30) limits statistical power and restricts the generalizability of the findings. Surgical or histopathological confirmation was not available for most cases, which may affect diagnostic accuracy when relying solely on imaging. Additionally, follow-up data were inconsistently available, preventing a comprehensive evaluation of long-term clinical outcomes. Multivariable analysis was not performed, limiting the independent assessment of potential confounding factors that may influence symptom development or complications.

## Conclusions

Effective imaging is critical for diagnosing colloid cysts and assessing their impact on CSF flow and ventricular dilatation. MRI, and in some cases CT, provides a comprehensive evaluation that aids in identifying hydrocephalus secondary to colloid cysts and supports timely clinical management. MRI not only confirms the presence and characteristics of the cyst but also helps distinguish symptomatic from asymptomatic cases. Signal characteristics, such as T1 hyperintensity suggestive of high cholesterol content and T2 hypointensity indicative of gelatinous consistency, are particularly relevant for surgical planning, as firmer cysts may present challenges during endoscopic removal.

Cyst size and location are pivotal in determining the need for intervention. Lesions larger than 10 mm more frequently obstruct CSF flow at the foramen of Monro, resulting in hydrocephalus. Imaging findings such as periventricular edema on FLAIR sequences further indicate elevated ICP and necessitate urgent surgical evaluation. The choice of surgical approach is often guided by imaging: smaller, non-adherent cysts are suitable for endoscopic resection, whereas larger or more complex cysts may require open microsurgical excision to achieve complete removal and minimize recurrence. Thus, imaging plays a central role not only in diagnosis but also in risk stratification and treatment planning.
